# Oxidative cell death in cancer: mechanisms and therapeutic opportunities

**DOI:** 10.1038/s41419-024-06939-5

**Published:** 2024-08-01

**Authors:** Xiaoqin An, Wenfeng Yu, Jinbao Liu, Daolin Tang, Li Yang, Xin Chen

**Affiliations:** 1 Department of Physiology, School of Basic Medical Sciences, Guizhou Medical University, Guiyang, Guizhou PR China; 2 Provincial Key Laboratory of Medical Molecular Biology, Guizhou Medical University, Guiyang, Guizhou PR China; 3 Key Laboratory of Biological Targeting Diagnosis, Therapy and Rehabilitation of Guangdong Higher Education Institutes, The Fifth Affiliated Hospital, Guangzhou Medical University, Guangzhou, Guangdong PR China; 4 Guangzhou Municipal and Guangdong Provincial Key Laboratory of Protein Modification and Degradation, State Key Laboratory of Respiratory Disease, School of Basic Medical Sciences, Guangzhou Medical University, Guangzhou, Guangdong PR China; 5 Department of Surgery, UT Southwestern Medical Center, Dallas, TX USA

**Keywords:** Cancer, Cell death

## Abstract

Reactive oxygen species (ROS) are highly reactive oxygen-containing molecules generated as natural byproducts during cellular processes, including metabolism. Under normal conditions, ROS play crucial roles in diverse cellular functions, including cell signaling and immune responses. However, a disturbance in the balance between ROS production and cellular antioxidant defenses can lead to an excessive ROS buildup, causing oxidative stress. This stress damages essential cellular components, including lipids, proteins, and DNA, potentially culminating in oxidative cell death. This form of cell death can take various forms, such as ferroptosis, apoptosis, necroptosis, pyroptosis, paraptosis, parthanatos, and oxeiptosis, each displaying distinct genetic, biochemical, and signaling characteristics. The investigation of oxidative cell death holds promise for the development of pharmacological agents that are used to prevent tumorigenesis or treat established cancer. Specifically, targeting key antioxidant proteins, such as SLC7A11, GCLC, GPX4, TXN, and TXNRD, represents an emerging approach for inducing oxidative cell death in cancer cells. This review provides a comprehensive summary of recent progress, opportunities, and challenges in targeting oxidative cell death for cancer therapy.

## Facts

- Multiple oxidative and antioxidant systems collaborate to influence cellular functions.
- An excessive buildup of ROS drives oxidative cell death.
- There are several manifestations of oxidative cell death, including ferroptosis, apoptosis, necroptosis, pyroptosis, paraptosis, parthanatos, and oxeiptosis.
- The induction of oxidative cell death emerges as a key strategy in the field of cancer therapeutics.

## Open questions

- How can we optimize the specificity of ROS-targeted agents for application across various cancer contexts, thereby improving precision in cancer treatment?
- How can we achieve significant progress in the clinical application of anti-cancer agents by modulating antioxidant systems?
- How can the distinct roles of ROS in driving various cell death modalities be distinguished?

## Introduction

The inexorable reality for all living entities is mortality, a fate shared by every cell within the human body. Cell death serves not only as a physiological mechanism controlling normal development and tissue balance, but also as a pathological process triggering organ dysfunction and causing local or systemic inflammation. Categorized on the basis of distinct biochemical processes and their susceptibility to intervention by pharmaceutical agents or genetic factors, modes of cell death can be broadly divided into two fundamental categories: accidental cell death (ACD) and regulated cell death (RCD) [1]. ACD represents an unregulated event, while RCD is controlled by various genes or proteins [1]. The list of RCD is expanding and includes apoptosis, ferroptosis, necroptosis, pyroptosis, paraptosis, parthanatos, oxeiptosis, alkaliptosis, cuproptosis, and disulfidptosis [2–5]. These RCD models have revealed associations with various human pathological conditions, providing potential insights for disease treatment.

Different modes of cell death are rooted in distinct cell signaling pathways and are often characterized by the convergence of these pathways. A quintessential example is the burgeoning body of evidence demonstrating that reactive oxygen species (ROS) can serve as triggers for diverse forms of cell death collectively known as oxidative cell death. ROS, which stem from aerobic metabolism, various stressors, or disruptions in antioxidant defenses, influence cellular fate by regulating their levels [6]. While moderate ROS levels are involved in a spectrum of signaling pathways that are vital for cell growth, differentiation, and progression, elevated ROS levels are potent triggers of cell death [7]. In cancer cells, heightened oxidative stress results from increased ROS production and/or compromised ROS-scavenging capacity [8]. Even a slight elevation in ROS levels within cancer cells relative to that in normal cells can surpass a critical threshold, inducing cancer cell death and suppressing tumor development [9]. Thus, agents that induce ROS generation hold the potential to be used in targeted strategies for eradicating malignancies.

This review discusses the origins of ROS, the impact of antioxidant systems on ROS dynamics, the main types and mechanisms of oxidative cell death, and the prospective use of small-molecular compounds or drugs to induce oxidative cell death as a tactic to counteract cancer.

## Overview of the production and regulation of ROS

**Fig. 1:**
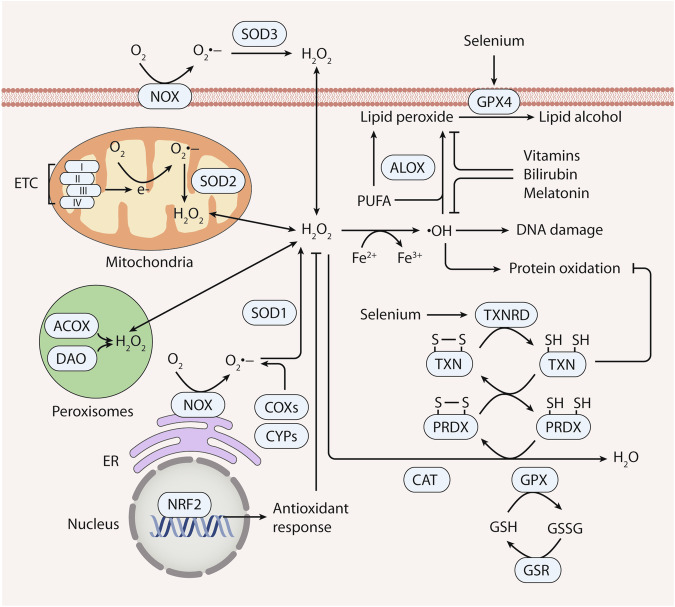
Overview of ROS generation and elimination. Reactive oxygen species (ROS) are labile oxygen-containing molecules primarily generated by the mitochondrial electron transport chain (ETC), peroxisomes, NADPH oxidase (NOX), lipoxygenase (ALOX), cyclooxygenases (COXs), and cytochrome P450s (CYPs). ROS elimination is facilitated by antioxidant systems, encompassing enzymatic antioxidants (e.g., SOD, CAT, GPX, and the thioredoxin [TXN]-thioredoxin reductase [TXNRD] system) and non-enzymatic antioxidants (e.g., glutathione [GSH], vitamins or analogs, selenium, and metabolites such as bilirubin and melatonin). Superoxide dismutase (SOD) transforms O_2_^•−^ into H_2_O_2_, subsequently reduced to H_2_O by catalase (CAT), glutathione peroxidase (GPX), or peroxiredoxins (PRDX). Among these, ALOX significantly contributes to lipid peroxidation, while GPX4, a selenocysteine-containing enzyme, quenches lipid peroxides. Central to the antioxidant network, TXNRD—a pivotal selenoprotein antioxidant—donates electrons to the TXN–PRDX axis. Moreover, the transcription factor NRF2 prominently regulates the antioxidant system, orchestrating the expression of genes crucial to antioxidant defense mechanisms.

ROS, which are highly reactive and short-lived molecules, readily engage with other cellular constituents, including lipids, proteins, and nucleic acids [10]. The intracellular ROS levels are meticulously governed by an intricate interplay of mechanisms governing both ROS generation and elimination (Fig. 1). In the following sections, we will delve into the sources and sites of ROS production, along with the enzymatic and non-enzymatic antioxidants involved in ROS-scavenging systems.

### ROS sources

ROS are unstable oxygen-containing molecules that include radical ROS (e.g., superoxide anions [O_2_^•−^], hydroxyl radicals [•OH], peroxyl radical [ROO•], alkoxyl radical [RO•], carbonate radical [CO_3_^•−^], hydroperoxyl radical [HO_2_•], nitric oxide [NO•], and nitrogen dioxide [NO_2_•]) and non-radical ROS (e.g., hydrogen peroxide [H_2_O_2_], peroxynitrite [ONOO^−^], hypochlorous acid [HOCl], singlet oxygen [^1^O_2_], ozone [O_3_], and nitrocarbonate [ONOOCO_2_^−^]) [11] (Table 1). The main sources of ROS under physiological conditions include the mitochondrial respiratory chain, peroxisomes, NADPH oxidase (NOX), and lipoxygenase (ALOX), and additional ROS-producing enzymes, such as cyclooxygenases (COXs) and cytochrome p450s (CYPs) [12, 13].

**Table 1:** Main types of ROS.

Category	ROS molecule	Notation	Main source	Scavenging systems
Radicals	Superoxide anions	O_2_^•−^	Mitochondrial respiratory chain, NOX, and peroxisomes	SOD and bilirubin
Radicals	Hydroxyl radicals	•OH	Reactions of H_2_O_2_ with O_2_ (Haber–Weiss), Fenton reaction	GSH, vitamins, and melatonin
Radicals	Peroxyl radical	ROO•	ALOX	GPX, various non-enzymatic antioxidants
Radicals	Alkoxyl radical	RO•	ALOX	Various non-enzymatic antioxidants
Radicals	Carbonate radical	CO_3_^•−^	SOD, XO	Various non-enzymatic antioxidants
Radicals	Nitric oxide	NO•	eNOS, iNOS, XO	Various non-enzymatic antioxidants
Radicals	Nitrogen dioxide	NO_2_•	MPO	Various non-enzymatic antioxidants
Radicals	Hydroperoxyl radical	HO_2_•	Reaction of •OH with H_2_O_2_	Various non-enzymatic antioxidants
Nonradicals	Hydrogen peroxide	H_2_O_2_	O_2_^•−^ dismutation by SOD, XO	GSH, CAT, GPX, TXN, TXNRD, and PRDX
Nonradicals	Peroxynitrite	ONOO^−^	MPO, ALXO, CYP	Various non-enzymatic antioxidants
Nonradicals	Hypochlorous acid	HOCl	MPO	Various non-enzymatic antioxidants
Nonradicals	Singlet oxygen	^1^O_2_	Photosensitization reactions	Various non-enzymatic antioxidants
Nonradicals	Ozone	O_3_	Interaction of ultraviolet radiation with O_2_	Various non-enzymatic antioxidants
Nonradicals	Nitrocarbonate	ONOOCO_2_^−^	Reaction of CO_2_ with ONOO^−^	Various non-enzymatic antioxidants

Within mammalian cells, mitochondria are the principal source of endogenous ROS [14]. The initial outcome of the mitochondrial respiratory chain is the generation of O_2_^•−^, which is formed at numerous mitochondrial locations, encompassing NADH:ubiquinone oxidoreductase (complex I), succinate dehydrogenase (complex II), ubiquinol:cytochrome c oxidoreductase (complex III), and mitochondrial glycerol 3-phosphate dehydrogenase [15]. Consequently, mitochondrial ROS production is associated with a spectrum of human pathological conditions or diseases, spanning inflammation, cancer, and neurodegenerative disorders [16]. In addition to mitochondria, peroxisomes also participate in ROS production. Peroxisomes produce ROS and reactive nitrogen species (RNS), such as H_2_O_2_, O_2_^•−^, •OH, NO•, and ONOO^−^ [17]. Moreover, peroxidative ROS levels depend on the caution of the antioxidant systems, which shield cells from oxidative harm, incorporate various peroxisomal enzymes, such as catalase (CAT), superoxide dismutase (SOD), glutathione peroxidase (GPX), and peroxiredoxins (PRDX), as well as low molecular weight non-enzymatic antioxidants [17]. Perturbations in peroxisomal activity that disturb redox homeostasis are implicated in the carcinogenesis of prostate cancer and hematological malignancy [18]. NOX, a vital contributor to ROS production, uses electrons from NADPH to generate O2^•–^. ALOX proteins are iron-containing enzymes that are crucial for driving lipid peroxidation through catalyzing the stereoselective oxygen insertion of polyunsaturated fatty acids (PUFAs), particularly arachidonic acid (AA) and adrenic acid. Dysregulation and aberrant activity of NOX and ALOX are implicated in a spectrum of diseases, including cancer. For instance, certain NOX members, such as NOX4, are overexpressed in various types of cancer (e.g., hepatocellular carcinoma and renal cell carcinoma) [19, 20]. The aberrant activation of ALOX5 is important for the proliferation and migration of breast cancer cells [21], whereas the absence of ALOX5 promotes the progression of bladder cancer by enabling the evasion of ferroptosis [22].

In certain cancer cells, such as those in lung cancer, heightened metabolic activity, particularly within mitochondria, is the primary driver of ROS generation [23]. This metabolic boost is often fueled by oncogenic signals, such as constitutively active mutant *KRAS* and *MYC* [24, 25]. Elevated ROS levels, especially in the early stages of cancer, can contribute to carcinogenesis by promoting genomic instability, mitogenic signaling pathways, and the NF-κB pathway, among others [26]. In advanced-stage tumors, cancer cells frequently exhibit numerous genetic alterations and increased oxidative stress [26]. This finding demonstrated the potential of these cells to be selectively targeted and eliminated through the pharmacological induction of ROS.

### ROS elimination

Antioxidant systems necessitate the presence of antioxidants to counterbalance free radicals and neutralize oxidants. These antioxidants are classified into enzymatic and non-enzymatic categories [27]. Enzymatic antioxidants include SOD, CAT, GPX, and the thioredoxin (TXN)–thioredoxin reductase (TXNRD) system. On the other hand, non-enzymatic antioxidants include glutathione (GSH), vitamins (such as vitamin C and vitamin E), selenium, and metabolites (including bilirubin and melatonin). Furthermore, the transcription factor NFE2 like bZIP transcription factor 2 (NFE2L2, best known as NRF2) is a pivotal regulator of the antioxidant system and controls the expression of genes central to antioxidant defense mechanisms [28]. In contrast, the transcription factor BTB domain and CNC homolog 1 (BACH1) can antagonize NRF2 by competing for binding to antioxidant response elements (AREs) in the promoter regions of target genes [29]. Thus, the balance between NRF2 and BACH1 activity determines the cellular response to oxidative stress in cancer [29]. In this section, we introduce the primary enzymatic and non-enzymatic antioxidants.

#### Enzymatic antioxidants

##### SOD

SOD catalyzes the conversion of O_2_^•−^ into oxygen and H_2_O_2_, thereby inhibiting the potential toxicity of O_2_^•−^. Three distinct SOD subtypes, namely, Cu/ZnSOD (encoded by the SOD1 gene), MnSOD (encoded by the SOD2 gene), and extracellular SOD (ecSOD, encoded by the SOD3 gene), have been identified and characterized in mammals [30]. Although O_2_^•−^ is not a strong oxidant, it is still potentially toxic. O_2_^•−^ oxidizes functional proteins, resulting in structural alterations, cluster degradation, and loss of enzyme activity [31]. Decreased SOD activity is correlated with heightened levels of oxidative damage, encompassing membrane lipid peroxidation, protein carbonylation, and DNA fragmentation [32]. SOD1 overexpression has been observed in tumor tissues, including those of the lung and breast, where it plays a crucial role in driving oncogene-driven cell proliferation [33, 34]. In addition, elevated levels of serum SOD1 might be linked to a higher risk of gastric cancer in humans [35]. Notably, targeting SOD could be a promising strategy for selectively killing cancer cells [36].

##### CAT

CAT is distributed throughout the human body and is notably expressed in organs, such as the liver, kidney, and red blood cells [37]. The CAT enzyme comprises four identical subunits, each weighing 62 kDa [38]. These subunits are endowed with four discrete domains, alongside an incorporated heme group [38]. As the principal antioxidant enzyme of peroxisomes, the catalytic role of CAT involves the conversion of H_2_O_2_ into H_2_O and oxygen (O_2_) [39]. CAT is overexpressed in multiple cancer types, including gastric cancer, colon cancer, melanoma, and leukemia [40–43]. Additionally, CAT protects tumor cells from ROS-induced apoptosis [44]. In contrast, pharmacologic inhibition of CAT induces apoptosis in cancer cell lines, such as lung and ovarian cancer [45].

##### GPX

GPX is assumed to play a pivotal role as an essential antioxidant enzyme, driving the reduction of H_2_O_2_ and organic hydroxides to their corresponding alcohols, with GSH serving as the reducing agent [46, 47]. The GPX family encompasses eight members, designated as GPX1-8, which collectively contribute to inhibiting oxidative stress and maintaining redox equilibrium [46]. However, each member of this family operates via a distinct mechanism and exhibits specific sites of action in maintaining redox homeostasis [46]. Among these, GPX4 has a unique capacity to selectively interact with the polar head of phospholipids, facilitating its association with bilayer membranes [47, 48]. By operating with GSH as its reducing substrate, GPX4 exhibits remarkable efficacy against diverse lipid peroxidation products, thereby blocking cell death induced by cytoplasmic or mitochondrial ROS and lipid peroxidation [48]. Pharmacological therapies targeting GPX4 are a promising strategy for inducing ferroptosis in cancer cells, including clear-cell carcinomas that resistant to conventional treatments [49].

##### TXN–TXNRD system

TXN and TXNRD together constitute a vital antioxidant defense system, which is instrumental in averting the excessive buildup of ROS within the body [50]. TXN, also referred to as TRX, acts as an enzyme that engages in the reduction of oxidized proteins through its redox-active site. This site features a distinctive and highly conserved motif housing two cysteine residues. TXN comprises two subtypes: TXN1, which is found in the cytosol and nucleus, and TXN2, which is localized within mitochondria [51]. Reduced TXN orchestrates the reduction in oxidized cysteines found in numerous proteins influenced by ROS. This process results in the oxidation of TXN itself, characterized by the formation of disulfide bonds between the sulfhydryl groups of cysteine residues [50]. In conjunction with its cofactor NADPH, TXNRD (also termed TrxR) catalyzes the disulfide reduction of TXN [50]. The upregulation of TXN and TXNRD in cancer cells enhances their proliferative and resistance to apoptosis, underscoring the TXN–TXNRD system as a promising target for therapeutic intervention [52, 53]. Therefore, inhibiting the function of TXN and TXNRD disrupts the redox balance in cancer cells, leading to heightened oxidative stress and subsequent cell death [54].

#### Non-enzymatic antioxidants

Alternative defense against ROS includes non-enzymatic antioxidants, such as GSH, vitamins or their analogs, selenium, and metabolites. GSH, generated from glutamic acid, cysteine, and glycine, is the most prevalent antioxidant in organisms [55]. GSH can undergo catalytic conversion to GSSG by GPX [46]. This transformation occurs simultaneously with the reduction of harmful peroxides into benign hydroxyl compounds or the facilitation of H_2_O_2_ decomposition [46]. This intricate process serves as an effective protective mechanism, guarding the structure and function of the cell membrane against damage caused by peroxides [46, 47]. Vitamins include non-enzymatic antioxidants, such as vitamin C and vitamin E [56]. Vitamin C can eliminate various oxygen free radicals, while vitamin E serves as a fat-soluble antioxidant that safeguards PUFAs within membranes from oxidation [57]. Selenium, a chemical element, is indispensable for the optimal operation of numerous physiological processes within living organisms, including humans [58]. The essential impact of selenium is largely realized through its incorporation into the foundational family of selenoproteins, such as GPX and TXNRD [59]. Furthermore, several metabolites, such as bilirubin and melatonin, have antioxidant effects [60, 61]. These non-enzymatic antioxidants, whether from diet or synthesized for therapy, vary in effectiveness against human cancer, showing both successes and failures in use [62–64]. However, accurately forecasting cellular responses to particular antioxidants based on current research poses a challenge. For instance, the administration of vitamin C or NAC promotes angiogenesis in lung tumor xenografts, while also enhancing cancer immunotherapy in colorectal cancer, breast cancer, and pancreatic cancer [64, 65]. Therefore, evaluating the potential anti-cancer effects of antioxidants on an individualized basis is crucial.

#### Core transcription factor

The transcription factor NRF2 is a key regulator of the antioxidant system by controlling the expression of genes vital for cellular defense against diverse detrimental stimuli [28]. NRF2 orchestrates the upregulation of several antioxidant genes reliant on GSH, including GSR, solute carrier family 7 member 11 (SLC7A11), glutamate–cysteine ligase catalytic subunit (GCLC), and glutamate–cysteine ligase modifier subunit, as well as metabolic detoxification genes such as aldehyde dehydrogenase 1 family member A1 and aldo–keto reductase family 1 member C1 in cancer cells [66]. These genes, under the influence of NRF2, fortify the cellular shield against ROS by binding to AREs, which contribute to mechanisms of ROS-mediated tumor chemoresistance [67].

A key regulator of NRF2 activity is kelch-like ECH-associated protein 1 (KEAP1), which acts as a ROS sensor and principal suppressor of NRF2. When cysteine residues in KEAP1 undergo redox oxidation, NRF2 interacts with the Cul3/RING-box protein complex, ultimately leading to NRF2 ubiquitination and subsequent degradation via proteasomes. The role of NRF2 in oncogenesis is intricate [68]. On the one hand, its activation can promote cancer progression, invasion, metastasis, and resistance to chemotherapeutic agents [68]. On the other hand, NRF2 holds the potential to prevent cancer initiation caused by oxidative stress [68]. The role of NRF2 has been confirmed beyond initial assumptions, presenting both challenges and opportunities in cancer treatment [69]. Nevertheless, cancer cells may employ the NRF2 transcription factor to counteract the excessive production of ROS [70]. Consequently, the reliance of tumors on the NRF2 antioxidant systems presents potential targets for cancer treatment. New strategies and prospects for effective cancer therapy can be explored for targeting NRF2.

## The mechanism of oxidative cell death

Excessive ROS can directly damage organelles, including the plasma membrane, ultimately leading to cell death [71]. In addition to common membrane repair mechanisms, such as the endosomal sorting complex needed for transport-III [72, 73], various antioxidant enzymes or proteins play a context-dependent role in selectively inhibiting oxidative cell death in cancer cells. ROS can initiate different modes of cell death through their oxidative effects on specific redox-sensitive proteins. For instance, ROS-mediated modification of KEAP1 precipitates oxeiptosis, while ROS-induced DNA damage initiates parthanatos [74, 75]. Additionally, ROS-mediated peroxidation of lipids triggers ferroptosis by inducing oxidative damage to PUFAs [76]. However, the types of cell death can vary depending on the cell type and context, adding to the complexity of the process. Below, we introduce the different types and their intricate mechanisms of oxidative cell death.

### Ferroptosis

Ferroptosis, an iron-dependent form of cell death, is primarily triggered by the accumulation of toxic lipids, especially lipid hydroperoxides [77]. Inhibition of the system xc^−^–GSH–GPX4 axis can predispose cells to ferroptosis, while the primary initiation of ferroptosis typically occurs through the accumulation of toxic lipids, as discussed later in the section. System xc^−^ serves as an amino acid reverse transporter that orchestrates the exchange of extracellular cystine and intracellular glutamate across the cell membrane [77]. The pivotal role of cysteine (the reduced form of cystine) as the rate-limiting substrate for GSH synthesis becomes evident here; system xc^−^ inhibition leads to a depletion of the vital antioxidant GSH. GSH, in turn, acts as a cofactor for GPX4, a specialized enzyme responsible for preventing lipid peroxidation [78]. Consequently, the suppression of system xc^−^ translates to a decrease in GPX4 function, precipitating ferroptotic cell death. Notably, FSP1 (also known as AIFM2) and DHODH inhibit ferroptotic cell death in a GPX4-independent manner. FSP1 reduces CoQ10 to CoQH2 [79–81], while also engaging in vitamin K reduction and promoting membrane repair, collectively mediating its anti-ferroptotic activity in cancer cells [82–84]. As a central transcription factor, NRF2 influences this cell death mode by inducing a variety of genes including SLC7A11, GPX4, FSP1, NQO1, HMOX1, ferritin heavy chain 1 (FTH1), HECT and RLD domain-containing E3 ubiquitin protein ligase 2, and vesicle-associated membrane protein 8 in hepatocellular carcinoma and ovarian cancer cells [85, 86]. Thus, NRF2 prevents ferroptosis by inducing the expression of genes involved in both GPX4-dependent and GPX4-independent pathways. In contrast, BACH1 exerts its pro-ferroptotic role by modulating the expression of genes (e.g., FTH1) involved in key regulatory pathways of ferroptosis in cancer cells [87, 88].

**Fig. 2:**
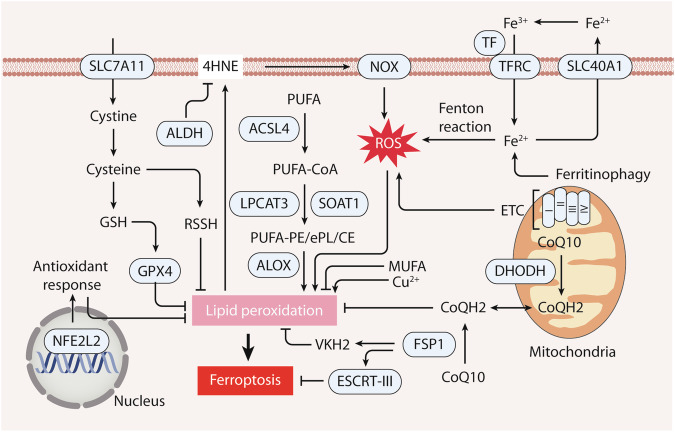
The role of ROS in ferroptosis. Ferroptosis, an iron-dependent form of cell death, is initiated by ROS-mediated lipid hydroperoxides. ROS primarily stem from the mitochondrial electron transport chain (ETC), NADPH oxidase (NOX), and Fe^2+^-mediated Fenton reactions. The transporter system xc^−^ relies on SLC7A11, a key subunit, to uptake extracellular cystine, a rate-limiting substrate for glutathione (GSH) synthesis. GSH, in turn, cofunctions with GPX4, a critical enzyme quenching lipid peroxidation, and aids in generating antioxidant hydropersulfides (RSSH) from cysteine. GSH-independent ferroptosis suppressors—FSP1 and DHODH—participate in CoQ10 to CoQH2 conversion, alongside FSP1’s roles in vitamin K reduction and membrane repair. The pivotal transcription factor NRF2 orchestrates gene expression to counteract ferroptosis. Fatty acids influence ferroptosis, with polyunsaturated fatty acids (PUFA) promoting it and monounsaturated fatty acids (MUFA) inhibiting it. Enzymes like ACSL4, LPCAT3, and SOAT1 mediate PUFA-CoA formation and subsequent esterification, while 4-hydroxynonenal (4HNE) from lipid hydroperoxides can activate cellular damage through the NOX pathway. Aldehyde dehydrogenase (ALDH) clears 4HNE, limiting ferroptosis. Iron’s import via transferrin (TF) and transferrin receptor (TFRC), as well as its export by SLC40A1/ferroportin, tightly regulates ferroptosis. Ferritinophagy, the autophagic degradation of ferritin, increases cytoplasmic Fe^2+^ levels, triggering ROS generation. Copper ions, along with iron, significantly contribute to initiating lipid peroxidation.

ROS-mediated lipid peroxidation is a hallmark of ferroptosis (Fig. 2). Since mitochondria represent the primary source of ROS, distinct metabolic activities within these organelles influence the initiation of ferroptosis in breast and prostate cancer [89]. Specifically, the leakage of electrons from mitochondrial ETC complexes gives rise to ROS, which can subsequently react with ferrous ions (Fe^2+^) to generate •OH radicals [89]. In turn, these radicals extract hydrogen from PUFAs, resulting in the formation of PUFA radicals (PUFA•) [47]. These highly reactive carbon-centered radicals then swiftly engage with oxygen, producing PUFA peroxyradicals (PUFA-OO•), and culminating in the generation of PUFA hydroperoxides (PUFA-OOH) [47]. Thus, the production of mitochondrial ROS contributes to the propagation of lipid peroxidation, potentially leading to ferroptosis.

The Lands’ cycle involves the removal and addition of fatty acids to phospholipids, which regulates development, immunity, inflammation, and other cellular functions [90]. The phospholipid acyl chain remodeling (Lands’ cycle) is essential for facilitating ferroptosis through the enrichment of membranes with PUFA [91]. Central to this process in ferroptosis is acyl-CoA synthetase long chain member 4 (ACSL4), which catalyzes the formation of arachidonic acid acyl-coA derivatives [92–95]. Subsequently, lysophosphatidylcholine acyltransferase 3 (LPCAT3) esterifies these derivatives with phosphatidylethanolamine (AA-PE), forming a crucial intermediate [92–94]. This AA-PE intermediate is further oxidized by ALOX enzymes, resulting in the generation of lipid hydroperoxides and the eventual induction of ferroptosis [96]. The NOX family contributes to ROS generation and subsequent ferroptosis in ovarian and colorectal cancer cells [97–99]. In addition, peroxisome-driven ePL biosynthesis can foster lipid peroxidation and ferroptosis in ovarian and renal cancer cells [100, 101]. Alternatively, the suppression of the lipid flippase solute carrier family 47 member 1 (SLC47A1) enhances ferroptosis by favoring the ACSL4–sterol O-acyltransferase 1 (SOAT1) pathway over the ACSL4–LPCAT3 pathway, leading to the production of PUFA cholesterol esters in pancreatic cancer cells [102]. In contrast, the antioxidant enzyme GPX4, along with antioxidants such as vitamin E, inhibits lipid peroxide-mediated damage [94].

Fenton reaction refers to a set of chemical reactions involving H_2_O_2_ and transition metal ions, typically Fe^2+^, which generates •OH, one of the most reactive ROS. The general reaction can be represented as Fe^2+^ + H_2_O_2_→Fe^3+^ + •OH + OH^−^. In the ferroptotic process, iron plays a pivotal role in several processes, including Fenton reaction-driven ROS production and the activation of key enzymes involved in lipid peroxidation, such as ALOX, NOX, and mitochondrial complexes I and III [103]. The generation of •OH provokes the oxidation of PUFAs and thereby initiates the cascade leading to ferroptosis. In contrast, the buildup of lipid ROS and the consequent onset of ferroptosis can be hindered through intervention with iron-chelating agents (e.g., deferoxamine) and lipophilic antioxidants (e.g., ferrostatin-1 and liproxstatin-1) [77]. Maintaining iron levels within cells is a finely tuned process regulated by the orchestrated interplay of various factors during ferroptosis [103]. The import of iron into cells is facilitated by transferrin (TF), which binds with the transferrin receptor (TFRC) located on the plasma membrane. To curtail the unrestricted diffusion of iron, most intracellular Fe^2+^ is sequestered within ferritin under physiological conditions. Among the key players in iron homeostasis, solute carrier family 40 member 1 (SLC40A1, also known as ferroportin) is the only known iron exporter in mammals, pivotal for orchestrating cellular iron dynamics in ferroptotic pancreatic cancer cells [104]. During ferroptosis, triggering stimuli can activate the process of ferritin degradation, termed ferritinophagy, or lead to the degradation of SLC40A1, thereby augmenting the levels of cytoplasmic Fe^2+^ within unstable iron pools in cancer cells [104–106]. This increase in labile iron pools prompts the generation of a substantial quantity of ROS.

Collectively, ferroptosis, as a form of oxidative cell death, is strongly context-dependent and requires an in-depth exploration of its underlying mechanisms [76]. This complexity underscores the need for rigorous investigation into the role of various antioxidant systems within experimental models. Given the intricate interplay between different cellular components, organelles, and pathways that regulate ferroptosis [107], it is imperative to consider the broader cellular context when evaluating ferroptosis triggers, modulators, and potential therapeutic interventions.

### Apoptosis

Apoptosis is an extensively studied form of RCD that is orchestrated through the activation of caspases, protein cleavage, and the formation of apoptotic bodies. Morphologically, apoptosis is characterized by cell shrinkage, chromatin condensation, and the emergence of apoptotic bodies through cellular fragmentation [108]. Apoptotic pathways can be divided into two categories: extrinsic apoptotic pathways triggered by cell death receptors, and intrinsic apoptotic pathways involving dysfunctional mitochondria [109]. Further classification based on the activation of caspase proteases is possible by distinguishing between caspase-dependent and caspase-independent variants. Extrinsic apoptotic pathways are activated by interactions between cell surface exposed death-inducing ligands such as FAS ligands (FASL) and tumor necrosis factor (TNF), and their cognate receptors Fas cell surface death receptor (FAS) and TNF receptor (TNFR) [110, 111]. These death receptors bear a death domain (DD) that fosters intracellular protein‒protein interactions, which are pivotal in transmitting apoptosis-inducing signals. Fas-associated via death domain and TNFRSF1A associated via death domain (TRADD) facilitate the recruitment of initiator caspase 8 (CASP8) or caspase 10 (CASP10), resulting in the creation of a death-inducing signaling complex and subsequent activation of procaspase.

The mitochondrial apoptotic route responds to an array of stress signals, such as mitochondrial damage, ER stress, and oxidative stress. The BCL2 family encompasses ~20 members, which are pro-apoptotic or anti-apoptotic proteins. Pro-apoptotic elements, BCL2 associated X, apoptosis regulator (BAX) and BCL2 antagonist/killer 1 (BAK1), drive processes such as mitochondrial outer membrane permeabilization and the creation of the mitochondrial permeability transition pore (MPTP), thus regulating the formation of pores in the outer mitochondrial membrane [112]. A breach results in the release of apoptotic molecules, such as cytochrome c (CYCS), apoptosis-inducing factor mitochondria-associated 1 (AIFM1), and diablo IAP-binding mitochondrial protein (DIABLO/SMAC) [112]. The release of CYCS into the cytoplasm activates initiator caspase 9 (CASP9). Eventually, the sequential activation of caspase 3 (CASP3), a principal executor of apoptosis, is induced by the activation of initiator CASP8 or CASP10 via cell death receptor pathways, and CASP9 via mitochondrial pathways.

**Fig. 3:**
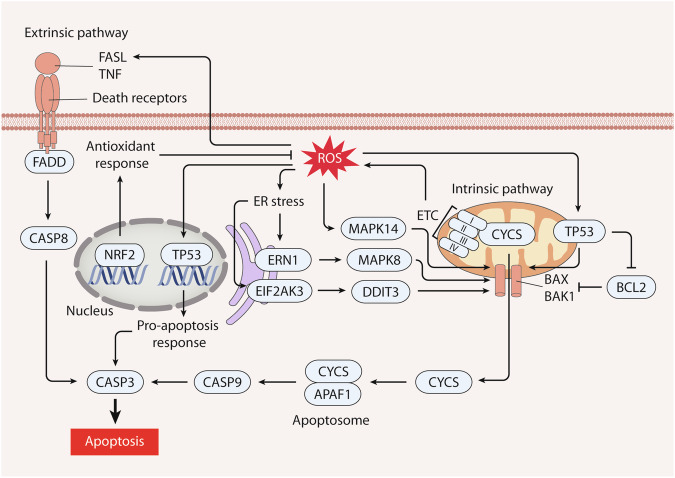
The role of ROS in apoptosis. Apoptotic pathways can be classified into two categories: extrinsic apoptotic pathways triggered by cell death receptors, and intrinsic apoptotic pathways involving mitochondria. Mitochondria serve as the primary intracellular source of ROS, emanating from electron leakage within the respiratory electron transport chain (ETC). The activation of mitogen-activated protein kinase 14 (MAPK14/p38) or ER stress by ROS influences this balance through anti-apoptotic BCL2 and pro-apoptotic BAX, which results in the release of apoptotic molecules, such as cytochrome c (CYCS). CYCS’s release into the cytoplasm activates initiator caspase 9 (CASP9). ROS may increase the expression of the tumor suppressor protein TP53, which fosters apoptosis not only through the transcriptional regulation of apoptosis-related genes, but also by translocating to the mitochondria. Mitochondrial TP53 interacts with BCL2 family proteins and amplifies mitochondrial membrane permeability independent of transcriptional mechanisms. In addition, ROS is involved in the extrinsic apoptotic pathway through enhancing the expression of both FAS and FASL genes. Eventually, the sequential activation of executor caspase 3 (CASP3) by CASP8 in the extrinsic pathways or CASP9 in the apoptotic pathways initiates apoptosis. On the contrary, NRF2 triggers the transcription of downstream antioxidant genes, effectively neutralizing ROS and mitigating apoptosis.

The initiation of apoptosis is closely related to ROS activity (Fig. 3). Mitochondria serve as the primary intracellular source of ROS and emanate from electron leakage within the respiratory ETC. The repercussions of these mitochondrial ROS include potential damage to neighboring structures, including mitochondrial DNA, which is vulnerable to oxidative harm. This leads to disruptions in the transcription of proteins vital to the mitochondrial ETC, thus inducing malfunction and hindering ATP synthesis, which in turn might escalate ROS generation [113]. An increase in oxidative stress also plays a role in MPTP opening, ultimately causing mitochondria-driven apoptosis. This can be observed in the modulation of BCL2 family protein levels by ROS, as heightened pro-apoptotic BAX and BAK1 levels accompanied by a decrease in anti-apoptotic BCL2 and BCL2-like 1 (BCL2L1/BCL-XL) expression are observed in squamous cell carcinoma cells [114]. Additionally, MCL1, another member of the BCL2 family, is involved in ROS generation via NOX4 during chemotherapy [115]. BCL2 inhibition by BH3 mimetic can trigger ROS generation to amplify apoptosis in cancer cells [116]. In contrast, NRF2 inhibits apoptosis through the detoxification of ROS in prostate cancer cells [117].

Furthermore, ROS-mediated ER stress is linked to the onset of apoptosis. The ER, which is responsible for essential cellular functions including protein folding and calcium storage/signaling, can experience disturbances in the folding environment, leading to the accumulation of misfolded proteins and subsequent ER stress. Under prolonged and severe ER stress, the unfolded protein response can eventually lead to apoptosis in pancreatic cancer cells [118]. In colon cancer cells, ER stress can stimulate ROS production, potentially intensifying apoptotic signals [119].

ROS-induced apoptosis is also correlated with increased expression or activity of the tumor suppressor protein p53 (TP53) [120]. Functioning as a transcription factor, TP53 orchestrates intrinsic apoptosis by dampening the presence of survival proteins such as MCL1, MYC, and BCL2, while elevating pro-apoptotic genes such as BAX in colorectal cancer cells [121, 122]. In this context, ROS-triggered TP53 activation heightens mitochondrial membrane permeability, leading to the release of pro-apoptotic factors from mitochondria.

Overall, these findings provide substantial evidence for the role of ROS in inducing apoptotic cell death, establishing that ROS are as a promising pathway in tumor therapy.

### Necroptosis

Necroptosis is a regulated form of cell death orchestrated by the interplay of key proteins, including receptor-interacting serine/threonine kinase 1 (RIPK1), receptor-interacting serine/threonine kinase 3 (RIPK3), and mixed lineage kinase domain-like pseudokinase (MLKL). The morphological characteristics of necroptosis include cell swelling, nuclear membrane dilation, chromatin condensation, cytoplasmic granulation, and plasma membrane rupture. These events lead to the release of cellular contents into the surrounding tissues, triggering an inflammatory response [123]. In different cell types, activation of necroptosis can be triggered by death receptors of the TNF family, including TNF receptor superfamily member 1A (TNFRSF1A/TNFR1), FAS, and TNF-related apoptosis-inducing ligand (TRAIL) receptors (TNF receptor superfamily member 10a [TNFRSF10A/TRAILR1] and TNF receptor superfamily member 10b [TNFRSF10B/TRAILR2]), that typically induce apoptosis. More specifically, TNF treatment induces apoptosis in F17 cells, but it provokes necroptosis in L-M cells [124]. Furthermore, additional receptors, such as TLR3, TLR4, and IFNAR1, play roles in initiating necroptosis, involving adapter molecules such as TIR domain-containing adapter molecule 1 (TICAM1, also known as TRIF) or RIHM-containing proteins Z-DNA binding protein 1 (ZBP1) [125, 126].

TNF-induced necroptosis is the most studied subtype. Interaction of TNF with TNFRSF1A leads to the recruitment of various proteins, including RIPK1, TRADD, baculoviral IAP repeat containing 2 (BIRC2/CIAP1) or baculoviral IAP repeat containing 3 (BIRC3/CIAP2), and TNF receptor-associated factor, which form a membrane-bound multimeric protein complex on the cytoplasmic side [123]. RIPK1 plays multiple roles, such as mediating nuclear factor-kappa B (NF-κB) activation, caspase-dependent apoptosis, and RIPK3-dependent necroptosis in response to activation signals. The presence of an RHIM domain in RIPK1 enables its binding to RIPK3 and subsequent RIPK3 activation through autophosphorylation in the cytoplasm. Upon phosphorylation by RIPK3, MLKL undergoes oligomerization and translocates to the plasma membrane, where it induces pore formation. CASP8 acts as a suppressor of necroptosis, and combined treatment with TNF and the pan-caspase inhibitor Z-VAD-FMK can activate necroptosis [127].

**Fig. 4:**
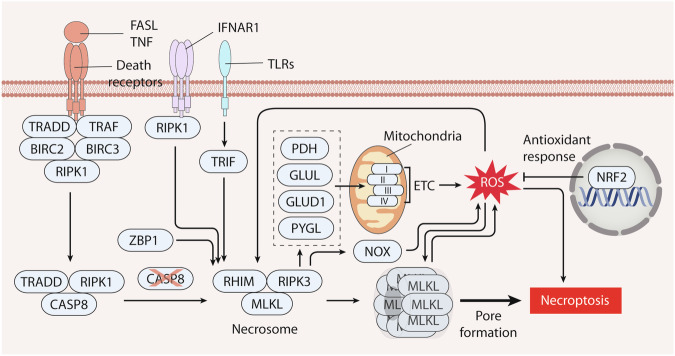
The role of ROS in necroptosis. Necroptosis is a regulated cell death orchestrated by receptor-interacting serine/threonine kinase 1 (RIPK1), receptor-interacting serine/threonine kinase 3 (RIPK3), and mixed lineage kinase domain-like pseudokinase (MLKL). Activation of receptors (e.g., TNFR1, TLR3, TLR4, and IFNAR1) prompts the recruitment of RHIM-containing proteins like RIPK1, TRIF, and ZBP1, and subsequent necrosome on the cytoplasmic side. Necroptosis is suppressed by caspase 8 (CASP8), and simultaneous treatment with TNF and caspase inhibitors can activate it. Subsequently, necrosomes form involving RIPK3 and MLKL in response to activation cues, which drives MLKL phosphorylation, oligomerization, and translocation to the plasma membrane for pore formation. ROS triggers RIPK1 autophosphorylation and MLKL activation. RIPK3 can also elevate ROS by stimulating mitochondrial metabolism and NADPH oxidase (NOX) activity. It enhances energy metabolism and mitochondrial ROS production through interactions with metabolic enzymes like pyruvate dehydrogenase complex (PDH), glutamate-ammonia ligase (GLUL), glutamate dehydrogenase 1 (GLUD1), and glycogen phosphorylase L (PYGL). Conversely, NRF2 induces transcription of antioxidant genes, mitigating ROS and ameliorating necroptosis.

Antioxidants that limit ROS production have been shown to inhibit TNF-induced necroptosis [128, 129], suggesting that ROS play a role in mediating necroptosis (Fig. 4). Indeed, ROS production may promote the activity of RIPK1 or MLKL in necroptosis induction. Mitochondrial ROS can activate the autophosphorylation of RIPK1 at Ser161 by oxidizing specific cysteines in RIPK1 in mouse fibroblast L929 cells [130]. ROS generation contributes to the activation of MLKL during necroptosis in lung cancer cells [131] and serves as a downstream event triggered by MLKL upon the induction of necroptosis in colon adenocarcinoma cells [132]. Additionally, RIPK3 can also enhance ROS production by activating mitochondrial metabolism or NOX activity in HT29 and Raji cancer cells [133]. Direct interactions between RIPK3 and enzymes, such as the pyruvate dehydrogenase complex (PDH), glutamate-ammonia ligase (GLUL), glutamate dehydrogenase 1 (GLUD1), and glycogen phosphorylase L (PYGL), can enhance energy metabolism and promote mitochondrial ROS production, thereby augmenting necroptosis in cervical adenocarcinoma and colon cancer cells [134]. Conversely, NRF2 induces the transcription of downstream genes such as HMOX1 and NQO1, neutralizing ROS and thereby alleviating necroptosis-mediated tissue injury [135].

In summary, ROS contribute to various aspects of necroptosis, including the initiation of signaling cascades, amplification of key protein activity, modulation of energy metabolism, activation of MLKL, and the establishment of a feedback loop. The balance between ROS production and antioxidant responses, as well as the cellular context, determines the impact of ROS on necroptosis and the subsequent cellular outcome.

### Pyroptosis

Pyroptosis is a distinct form of RCD characterized by the activation of inflammasomes and inflammation-associated caspases [136]. The classical inflammasome pathway involves multiple stages, including inflammasome assembly and activation, channel protein formation, and the maturation and secretion of pro-inflammatory cytokines (e.g., IL1B and IL18). Upon sensing environmental stress or cellular damage, the absent in melanoma 2 inflammasome (AIM2) and NLR family pyrin domain-containing 3 (NLRP3) inflammasome assemble and recruit the inflammasome adapter protein apoptosis-associated speck-like protein containing CARD, leading to the activation of pro-inflammatory caspases [137]. Caspase 1 (CASP1), when activated, cleaves gasdermin D (GSDMD), producing the pore-forming protein N-GSDMD, and also cleaves pro-IL1B and pro-IL18 into their mature forms [138]. A distinct nonclassical inflammasome pathway is triggered by direct binding of lipopolysaccharide (LPS) to caspase 11 (CASP11 in humans) or caspase 4/5 (CASP4/5 in mice), resulting in GSDMD cleavage [139]. Interestingly, CASP3 activation associated with apoptosis can also cleave gasdermin E (GSDME), bridging the connection between apoptosis and pyroptosis [140].

**Fig. 5:**
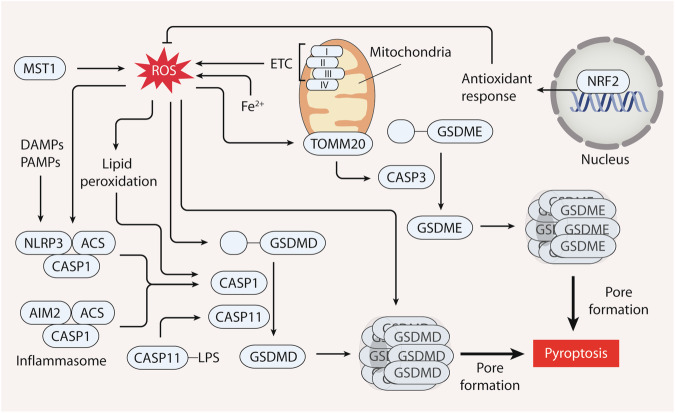
The role of ROS in pyroptosis. Pyroptosis is a mode of cell death marked by inflammasome activation and inflammation-associated caspases. Upon sensing damage-associated molecular pattern molecules (DAMPs) or pathogen-associated molecular pattern molecules (PAMPs), absent in melanoma 2 inflammasome (AIM2) and NLR family pyrin domain-containing 3 (NLRP3) inflammasomes assemble, which leads to caspase 1 (CASP1) activation. Alternatively, direct binding of lipopolysaccharide (LPS) to caspase 11 (CASP11) triggers CASP11 activation. Activated CASP1 or CASP11 cleaves gasdermin D (GSDMD), while CASP3 cleaves gasdermin E (GSDME), generating the pore-forming proteins N-GSDMD or N-GSDME, which induces cell death. ROS act as an upstream signal for NLRP3 inflammasome activation by upregulating pyroptosis-related genes like NLRP3 and CASP1. ROS or lipid peroxidation can also enhance GSDMD cleavage and CASP1 activation. Iron-induced ROS production activates caspase 3 (CASP3) via mitochondrial translocase of outer mitochondrial membrane 20 (TOMM20), triggering pyroptosis. Moreover, macrophage stimulating 1 (MST1) plays a role in pyroptosis regulation through promoting the production of ROS. In contrast, the pivotal regulator NRF2 curbs pyroptosis by reducing intracellular ROS levels.

In the context of pyroptosis, ROS play roles in the activation of the NLRP3 inflammasome and caspases (Fig. 5). ROS acts as upstream signals for NLRP3 inflammasome activation by upregulating the expression of key components, including NLRP3, pro-CASP1, and pro-IL1B [141]. Iron-induced ROS production has emerged as another influential factor in the initiation of pyroptosis, and has specific mechanisms involving mitochondrial translocase of outer mitochondrial membrane 20 (TOMM20)-mediated CASP3 activation in melanoma cells [142]. Similarly, lipid ROS can enhance GSDMD cleavage by activating CASP1 in macrophage [143]. Additionally, mitochondrial ROS-induced oxidation of GSDMD is a crucial mechanism facilitating GSDMD cleavage, which in turn drives NLRP3-dependent pyroptosis in macrophage [144]. This finding underscores the intricate interplay between iron homeostasis and mitochondrial function and the induction of pyroptotic cell death.

Furthermore, the core component of the Hippo pathway, macrophage stimulating 1 (MST1), is implicated in triggering pyroptosis. MST1 enhances ROS levels, thereby contributing to the initiation of pyroptosis in pancreatic cancer cells [145]. The Hippo pathway plays a fundamental role in controlling cancer cell growth, proliferation, and metastasis [146]. Targeting MST1 or its downstream effectors involved in ROS regulation might lead to novel strategies for modulating pyroptosis and its associated inflammatory consequences. These insights highlight the intricate interplay between ROS and the regulation of pyroptosis, where ROS serve as both signaling molecules that promote pyroptosis induction and a target for therapeutic intervention.

### Paraptosis

Paraptotic cells are characterized by unique features, including cytoplasmic vacuolation arising from extensive ER and mitochondrial dilatation [147]. Unlike other cell death modalities, paraptosis lacks the typical apoptotic hallmarks, such as DNA condensation, DNA breakage, membrane blistering, and apoptotic bodies. Unlike necrosis, paraptosis maintains membrane integrity. Unlike apoptosis, paraptosis does not hinge on caspase activity but frequently hinges on the activation of mitogen-activated protein kinases (MAPKs), such as MAPK8/JNK, MAPK14/p38, and mitogen-activated protein kinase kinase 2 (MAP2K2/MEK2) [148]. Paraptosis is often accompanied by protein misfolding, ER stress, disturbances in Ca^2+^ levels, and perturbations in redox equilibrium [149, 150].

**Fig. 6:**
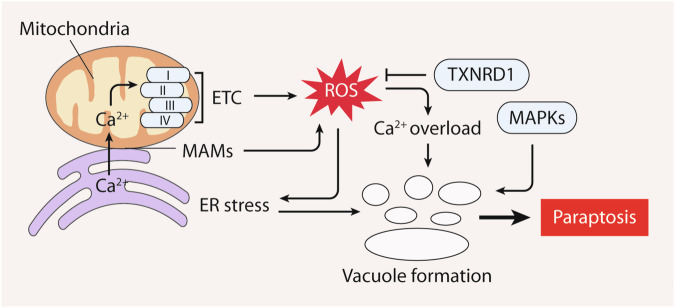
The role of ROS in paraptosis. Paraptosis is characterized by cytoplasmic vacuolation, resulting from extensive dilation of the endoplasmic reticulum (ER) and mitochondria. Vacuole formation in paraptosis necessitates the activation of mitogen-activated protein kinases (MAPKs). The onset of paraptosis is driven by ROS generation, initiating ER stress and Ca^2+^ overload. In contrast, thioredoxin reductase 1 (TXNRD1) critically curtails paraptosis by diminishing ROS production. Additionally, the interplay between mitochondria-associated ER membranes (MAMs) and the coordination of Ca^2+^ flux from the ER to mitochondria stand out as pivotal factors in inducing oxidative metabolic stress during paraptotic cell death.

Paraptosis essentially occurs as a result of excessive ROS generation (Fig. 6). Mitochondrial malfunction triggers ROS overproduction and an influx of Ca^2+^, resulting in vacuole formation, MAPK activation, and the initiation of paraptosis in prostate cancer cells [151]. TXNRD1 is a crucial regulator of paraptosis. Inhibiting TXNRD1 may drive ROS production, inciting ER stress and contributing to the paraptotic process in glioblastoma multiforme cells [152]. Moreover, the communication between MAMs and the coordination of Ca^2+^ flux from the ER to mitochondria are pivotal for triggering oxidative metabolic stress, a ROS surge, and an ensuing pro-paraptotic Ca^2+^ overload in breast cancer cells [153].

The intricate link between ROS production, Ca^2+^ imbalance, and MAPK activation exemplifies the intricate network that governs the initiation and execution of paraptosis. Further exploration of the regulatory mechanisms and functional implications of paraptosis will provide new insights into its significance in both physiological and pathological contexts.

### Parthanatos

Parthanatos is a distinct programmed cell death process driven by the activity of poly(ADP-ribose) polymerase 1 (PARP1) [75]. PARP1, a ribosyltransferase enzyme, plays a role in DNA repair by sensing single- and double-strand DNA breaks and facilitating the recruitment of repair machinery. However, excessive activation of PARP1 results in the excessive accumulation of poly(ADP-ribose) (PAR) molecules. The translocation of PAR from the nucleus to the mitochondria serves as a key event in initiating parthanatos. This translocation leads to the release of AIFM1 from mitochondria, which subsequently forms a complex with macrophage migration inhibitory factor (MIF) in the cytoplasm [154]. Cyclophosphamide, a nitrogen mustard, induces GPX4 degradation and activates AIFM1, leading to parthanatos in leukemia cells [155]. The translocated AIFM1–MIF complex then translocates back to the nucleus, where it contributes to chromatin condensation and DNA fragmentation, ultimately resulting in parthanatos-mediated cell death [154].

**Fig. 7:**
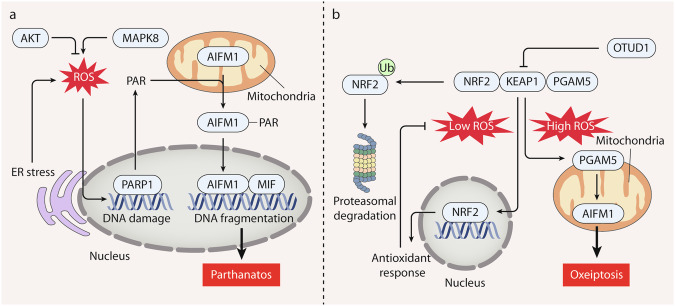
The role of ROS in parthanatos and oxeiptosis. **a** Parthanatos is a distinctive programmed cell death process driven by the enzymatic activity of poly(ADP-ribose) polymerase 1 (PARP1). This ribosyltransferase enzyme is crucial for DNA repair, detecting single- and double-strand DNA breaks and aiding in repair machinery recruitment. Oxidative stress acts as a central trigger, causing widespread DNA damage and excessive PARP1 activation. The resultant surplus of poly(ADP-ribose) (PAR) molecules leads to the liberation of apoptosis-inducing factor mitochondria-associated 1 (AIFM1) from mitochondria. AIFM1 then complexes with macrophage migration inhibitory factor (MIF), instigating parthanatos by orchestrating chromatin condensation and DNA fragmentation. The parthanatos process is influenced by mitogen-activated protein kinase 8 (MAPK8/JNK), AKT, and endoplasmic reticulum (ER) stress, which modulate intracellular ROS levels. **b** Oxeiptosis is distinguished by a notable ROS buildup, initiated via the kelch-like ECH-associated protein 1 (KEAP1)–PGAM family member 5-mitochondrial serine/threonine protein phosphatase (PGAM5)–apoptosis-inducing factor mitochondria-associated 1 (AIFM1/AIF) signaling cascade. KEAP1 acts as an inherent inhibitor of NRF2, leading to its proteasomal degradation. Under low ROS levels-induced oxidative stress, KEAP1 oxidizes and dissociates from NRF2, allowing NRF2 to translocate into the nucleus, thereby activating the transcription of numerous protective antioxidant genes. However, high ROS levels disrupt KEAP1’s interaction with another partner, PGAM5. This causes PGAM5 to relocate to the mitochondria, where it dephosphorylates AIFM1 and activates AIFM1, inducing oxeiptosis. The OTU deubiquitinase 1 (OTUD1) binds to and suppresses KEAP1’s ubiquitination, consequently inhibiting ROS-triggered oxeiptosis.

Oxidative stress is a central factor that triggers widespread DNA damage, which in turn leads to the overactivation of PARP1 and the initiation of parthanatos (Fig. 7a). For instance, exposure to H_2_O_2_ stimulates PARP1 activation and subsequent AIFM1 nuclear translocation in glioma cells [156]. Interventions such as the antioxidant NAC or the inhibition of MAPK8/JNK activity have been demonstrated to mitigate ROS-induced parthanatos in glioma cells [156], suggesting that MAPK8 activation contributes to parthanatos by enhancing intracellular ROS levels. Similarly, excessive ROS production is linked to parthanatos induction triggered by compounds, such as oxaliplatin and deoxypodophyllotoxin, in glioma and oral squamous cell carcinoma cells [157, 158]. Additionally, modulating signaling pathways, such as AKT pathways, influence parthanatos by altering intracellular ROS levels in colon cancer cells [159].

The intricate interplay between parthanatos and other cell death mechanisms represents a captivating area that demands further exploration. As we further our understanding of parthanatos, new possibilities for therapeutic interventions directed at modulating this cell death pathway are discovered, especially in the context of oxidative stress-associated diseases.

### Oxeiptosis

Oxeiptosis is a caspase-independent and non-inflammatory cell death pathway. This process is marked by a substantial accumulation of ROS, triggered through the activation of the KEAP1–PGAM family member 5, mitochondrial serine/threonine protein phosphatase (PGAM5)–AIFM1 signaling cascade (Fig. 7b) [74]. KEAP1, an intrinsic inhibitor of NRF2, plays a role in maintaining cellular redox homeostasis under low ROS levels [74, 160]. Increased oxidative stress induces high ROS levels and diminishes the interaction between KEAP1 and PGAM5, a mitochondrial serine–threonine phosphatase [74, 160]. This event causes the relocation of PGAM5 to the mitochondria [74]. Once within the mitochondria, PGAM5 dephosphorylates AIFM1 at Ser116, ultimately resulting in the induction of oxeiptosis in HeLa cervical cancer cells [74]. Oxeiptosis driven by AIFM1 does not necessitate the migration of AIFM1 from the mitochondria to the nucleus, a distinctive feature that sets oxeiptosis apart from parthanatos [74]. The suppression of K63-ubiquitination of KEAP1 is mediated by OTU deubiquitinase 1 (OTUD1), which may inhibit ROS-induced oxeiptosis in kidney cancer cells [161]. However, the full extent of the implications of oxeiptosis in diseases has not been fully elucidated. Despite being a relatively investigated oxidative cell death mechanism, much about its role in various conditions remains to be elucidated.

Depending on the type of tumor cells and the surrounding environment, oxidative cell death can occur in various forms. Ferroptosis relies primarily on ROS-induced lipid peroxidation, whereas other forms of cell death often result from ROS-induced alterations in protein or DNA function. The potential induction of other types of cell death by ROS, such as alkaliptosis, cuproptosis, and disulfidptosis, requires further investigation. Traditional cancer therapies mainly aim to induce apoptosis in cancer cells. However, it is widely recognized that a significant portion of cancer cells develop resistance to apoptosis. Fortunately, the identification and exploration of non-apoptotic cell death pathways, such as ferroptosis pathways, have opened up new opportunities for therapeutic interventions. Therefore, revealing the crosstalk and additional key regulators of oxidative cell death pathways is crucial for identifying new targets for drug development and screening.

### Autophagy in oxidative cell death

Autophagy is a cellular recycling system that is pivotal for breaking down and removing damaged or unnecessary proteins, organelles, and cellular debris. This process maintains cellular homeostasis by allowing cells to get rid of waste and reuse molecules for energy metabolism and material synthesis [162]. ROS can induce autophagy through several mechanisms, including direct activation of AMP-activated protein kinase or inhibition of the mammalian target of rapamycin [163, 164]. Additionally, ROS can directly oxidize and modify key autophagy modulators, such as ATG4, facilitating the formation of autophagosomes [165]. ROS-induced cellular stress can lead to misfolded and damaged organelles, prompting cells to activate autophagy to eliminate these harmful components and restore homeostasis [166, 167]. Overall, this ROS-induced autophagy serves as a crucial response to oxidative stress, enabling cells to adapt to adverse conditions and enhance survival.

The role of autophagy in oxidative cell death is context-dependent and can vary based on the specific cellular conditions and signaling pathways involved. Generally, autophagy can inhibit the initiation of apoptosis. ROS-induced autophagy prevents DNA damage- or TP53-mediated apoptotic cell death in colorectal cancer and cervical carcinoma [168, 169]. Similarly, autophagy plays a cytoprotective role in oxidative cell death in ovarian cancer and non-small cell lung cancer [170, 171]. Autophagy can inhibit apoptosis by selectively removing damaged mitochondria and pro-apoptotic proteins [172]. Recent studies have shed light on the essential role of autophagy in regulating ferroptotic cell death in cancer cells [173, 174]. This involves the selective degradation of anti-ferroptosis proteins or organelles, such as lipid droplets, GPX4, ferritin, SLC40A1, CDH2, and BMAL1 [105, 106, 175–180]. In contrast, reticulophagy, selective autophagy of the endoplasmic reticulum, exhibits a protective function against ferroptotic events in hepatocellular carcinoma [181]. The autophagy pathway also plays a complex role in tumor immunity and therapy [182]. Understanding the mechanisms by which autophagy regulates oxidative cell death may provide new therapeutic strategies against cancer.

## Oxidative cell death in tumor therapy

**Fig. 8:**
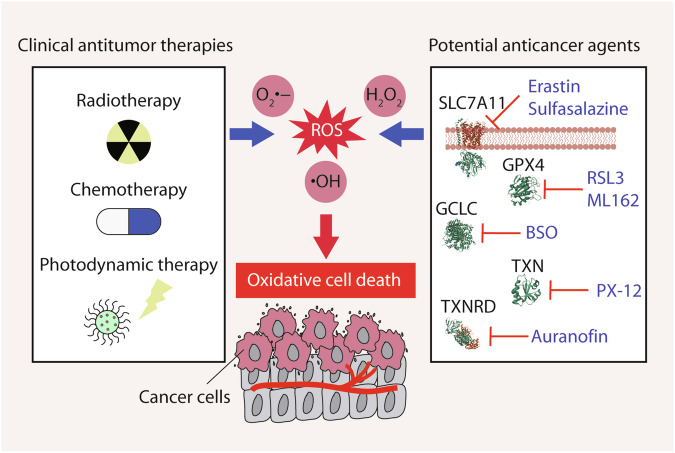
Strategies to inducing oxidative cell death in cancer. Approaches that promote the generation of ROS to trigger oxidative cell death hold great promise in anti-cancer therapeutics. Conventional anti-tumor treatments such as radiotherapy, chemotherapy, and photodynamic therapy, can leverage the elevation of ROS levels within cancer cells, leading to damage to the malignancy. Furthermore, potential anti-cancer agents targeting specific components of the antioxidant system, such as SLC7A11, GCLC, GPX4, TXN, and TXNRD, have the potential to selectively eliminate cancer cells.

ROS play dual roles in tumor development [183–185]. ROS can promote cell proliferation, DNA damage, and inflammation, driving malignant transformation. Conversely, elevated ROS can induce oxidative cell death in cancer cells. Therefore, strategies to increase ROS levels for cancer cell death, especially in advanced stages, are promising (Fig. 8). Numerous studies have highlighted the cytotoxic effects of ROS inducers on cancer cells by promoting oxidative cell death (Table 2). In the following discussion, we will emphasize clinical anti-tumor therapies that disrupt ROS homeostasis, along with potential anti-cancer agents targeting antioxidant enzymes.

**Table 2:** ROS inducers for cancer treatment.

Compound	Target	Types of cell death	Cancer types	Stage of development	Ref
Sorafenib	SLC7A11	Ferroptosis	Liver cancer	Approved anti-cancer drug	[206]
Sulfasalazine	SLC7A11	Ferroptosis	Pancreatic cancer, lung cancer	Approved antibiotics	[255]
Erastin	SLC7A11	Ferroptosis	Fibrosarcoma, lung cancer	Preclinical	[77]
IFNG	SLC7A11	Ferroptosis	Fibrosarcoma, ovarian cancer	Approved immunomodulatory drug	[256]
FIN56	GPX4	Ferroptosis	Bladder cancer	Preclinical	[257]
ML162	GPX4	Ferroptosis	Fibrosarcoma	Preclinical	[258]
ML210	GPX4	Ferroptosis	Fibrosarcoma	Preclinical	[258]
RSL3	GPX4	Ferroptosis	Lung cancer, colorectal cancer	Preclinical	[259]
N6F11	GPX4	Ferroptosis	Pancreatic cancer	Preclinical	[223]
PdPT	GPX4	Apoptosis and ferroptosis	Lung cancer	Preclinical	[260]
Plumbagin	GPX4	Apoptosis	Liver cancer	Preclinical	[221]
Buthionine sulfoximine (BSO)	GCLC	Apoptosis and ferroptosis	Ovarian cancer, breast cancer, melanoma	Phase I (NCT00002730)	[78]
PX-12	TXN	Apoptosis	Lung cancer, liver cancer	Phase II (NCT00417287)	[226, 261]
PMX464	TXN	Apoptosis	Colorectal cancer	Preclinical	[262]
Ferroptocide	TXN	Ferroptosis	Lung cancer, colorectal cancer	Preclinical	[231]
Auranofin	TXNRD	Apoptosis and paraptosis	Lung cancer, breast cancer	Approved anti-rheumatoid arthritis drug	[233, 234]
Piperlongumine	TXNRD	Apoptosis	Liver cancer	Preclinical	[263]
WZ26	TXNRD	Apoptosis	Colon cancer	Preclinical	[264]
Diffractaic acid	TXNRD	Apoptosis	Breast cancer	Preclinical	[265]
Thimerosal	TXNRD	Apoptosis	Lung cancer	Preclinical	[266]
Shikonin	TXNRD	Apoptosis and necroptosis	Glioma, breast cancer	Preclinical	[267, 268]
B63	TXNRD	Paraptosis	Gastric cancer	Preclinical	[269]
Jolkinolide B	TXNRD	Paraptosis	Bladder cancer	Preclinical	[270]
Nitrovin	TXNRD	Paraptosis	Colon cancer, bladder cancer	Preclinical	[152]
2-Methoxyestradiol	SOD1	Apoptosis	Prostate cancer, leukemia	Phase II (NCT00592579)	[271]
ATN-224	SOD1	Apoptosis	Epidermoid carcinoma	Phase II (NCT00405574)	[272]
Arsenic trioxide (As_2_O_3_)	ROS	Apoptosis	Leukemia, myeloma	Approved anti-cancer drug	[273]
Bortezomib	ROS	Apoptosis	Multiple myeloma, lung cancer	Approved anti-cancer drug	[274]
Cisplatin	ROS	Apoptosis and ferroptosis	Esophageal adenocarcinoma	Approved anti-cancer drug	[194]
Disulfiram	ROS	Apoptosis	Breast cancer	Approved anti-alcoholism drug	[275]
5-Fluorouracil (5-FU)	ROS	Apoptosis	Melanoma, colorectal cancer	Approved anti-cancer drug	[276]
Lanperisone	ROS	Non-apoptosis	Lung cancer	Approved muscle relaxant	[277]
Imexon	ROS	Apoptosis	Pancreatic cancer	Phase II (NCT00637247)	[278]
Nelfinavir	ROS	Apoptosis	Cervical cancer	Approved anti-HIV drug	[279]
Withaferin A	ROS	Paraptosis	Breast cancer	Phase II (NCT05610735)	[280]
Neobavaisoflavone	ROS	Pyroptosis	Liver cancer	Preclinical	[281]
Oxaliplatin	ROS	Parthanatos	Oral squamous	Approved anti-cancer drug	[158]
Sanguinarine	ROS	Oxeiptosis	Colorectal cancer	Preclinical	[282]
Auriculasin	ROS	Apoptosis, ferroptosis, and oxeiptosis	Colorectal cancer	Preclinical	[283]

### Clinical anti-tumor therapies that disrupt ROS homeostasis

Conventional anti-tumor therapies, such as radiotherapy and chemotherapy, as well as emerging strategies such as photodynamic therapy, aim to deliberately increase ROS levels to selectively target and eliminate cancer cells.

Ionizing radiation generates free radicals that exhibit high reactivity toward cellular macromolecules, including DNA, lipids, and proteins. Ionizing radiation leads to genetic instability, ultimately resulting in cancer cell apoptosis. Intriguingly, recent research highlights that ionizing radiation can trigger ferroptosis in certain cancer cells by amplifying lipid peroxidation [186, 187]. Ionizing radiation can modulate the expression of ACSL4 or SLC7A11[186–188]. Moreover, ionizing radiation can induce pyroptosis in cancer cells expressing the GSDME protein, such as those found in lung, liver, breast, and glioma cancers [189].

An increase in ROS production within cancer cells contributes significantly to the anti-cancer effects of various conventional chemotherapeutic drugs. Arsenic trioxide (As_2_O_3_) is an effective therapeutic for relapsed or refractory acute promyelocytic leukemia. As_2_O_3_ induces ROS generation by disrupting the mitochondrial ETC, impairing mitochondrial membrane potentials, and depleting GSH in HeLa and Calu-6 cancer cells [190, 191]. This induction of oxidative stress by As_2_O_3_ can activate a range of cell death pathways, including pathways related to apoptosis, necroptosis, pyroptosis, and ferroptosis [192, 193]. Likewise, several other chemotherapeutic agents, such as platinum complexes (e.g., cisplatin) [194] and sorafenib [195], enhance mitochondrial ROS production or hinder the antioxidant system, thereby causing heightened ROS levels within cancer cells.

Photodynamic therapy, a photochemical-based treatment approach, exploits the generation of chemical damage to eliminate tumor cells through the excitation of a photosensitizer. This therapy induces ROS generation by triggering direct photochemical reactions, inducing ER stress, or modulating the mitochondrial membrane potential [196]. Photodynamic therapy is capable of promoting various modes of cancer cell death, including apoptosis, paraptosis, and necroptosis [197, 198]. This approach is localized, minimizing damage to healthy tissue. Additionally, photodynamic therapy enhances the anti-tumor immune response, augmenting the activation of tumor-specific immune cells [199].

While these therapies target oxidative cell death and ROS balance in cancer cells, their effectiveness can vary due to factors such as tumor type, stage, and patient-specific characteristics. Balancing selective cancer cell killing with minimal harm to healthy cells remains a challenge in developing these therapies.

### Potential anti-cancer agents that target the antioxidant enzymes

In addition to traditional chemotherapeutic medications, certain agents can either target the antioxidant system or enhance the generation of ROS, potentially leading to cancer cell death. In this context, we primarily emphasize agents that target specific components of the antioxidant system, including SLC7A11, GCLC, GPX4, TXN, and TXNRD.

#### SLC7A11 inhibitor

SLC7A11, the catalytic subunit of system xc^−^, functions as a transporter responsible for cysteine influx into cells, a process pivotal for the survival and growth of cancer cells. Given the heavy reliance of many cancer cells on the transport activity of system xc^−^, this component has emerged as a promising target for the development of anti-cancer drugs. Inhibitors of SLC7A11 suppress cystine uptake, leading to a depletion of GSH and resulting in cell death mechanisms such as apoptosis and ferroptosis [200, 201].

Among these inhibitors, sulfasalazine, an FDA-approved drug commonly employed for treating inflammatory conditions, is one of the most widely used SLC7A11 inhibitors in laboratory settings. Sulfasalazine-mediated GSH depletion inhibits sarcoma cell growth both in vitro and in preclinical mouse models [202]. Sulfasalazine acts as a competitive inhibitor of SLC7A11 by interacting with the backbone of TM1a and R396 as well as Y240, Y244, and Y444 of SLC7A11 [203]. Clinical trials involving sulfasalazine combined with radiotherapy for 12 patients with newly diagnosed glioblastoma (NCT 04205357) have shown no significant impact on progression-free survival and overall survival of patients [204]. Ongoing clinical trials are investigating the anti-cancer potential of sulfasalazine in metastatic colorectal cancer (NCT06134388), breast cancer (NCT03847311), and acute myeloid leukemia (NCT05580861). Notably, sulfasalazine has poor oral bioavailability, estimated at 3–12% [205]. This is partly due to the ABCG2 efflux pump, which transports sulfasalazine out of cells, reducing its absorption and effectiveness [205]. Therefore, developing a high-bioavailability formulation of sulfasalazine could potentially enhance the effectiveness of cancer treatment.

Another notable contender is erastin and its analog, imidazole ketone erastin, which serve as potent ferroptosis inducers, the inhibitory effect of which exceeds that of sulfasalazine by more than 2000 times in HT-1080 and Calu-1 cancer cells [206]. These agents interact with hydrophobic pockets in SLC7A11, and the chlorophenoxy group binds to TM1a, TM6b, and TM7, while the quinazolinol group binds to TM5 and TM8 [207]. Although erastin and its derivatives show promising anti-tumor effects in vitro, they have not yet progressed to clinical trials. Several FDA-approved anti-cancer drugs, including sorafenib, can induce ferroptosis by inhibiting SLC7A11 activity, even though they also elicit apoptosis in preclinical studies [195]. Adding to the complexity, interferon-gamma (IFNG/IFN-γ), which is released from CD8^+^ T cells, downregulates the expression of SLC7A11, effectively promoting ferroptosis induction in HT-1080 and B16 cancer cells [208]. This finding suggests that IFNG functions as an endogenous inhibitor of SLC7A11, enhancing our understanding of the interplay between the immune response and ferroptosis in the context of cancer [209].

#### GCLC inhibitor

GSH is implicated in conferring resistance to a variety of anti-cancer drugs in cancer cells [210]. Depleting GSH levels can exert detrimental effects on cancer cells, potentially augmenting the efficacy of chemotherapy and ionizing radiation treatments [211]. Buthionine sulfoximine (BSO) is an irreversible inhibitor of GCLC, the rate-limiting enzyme responsible for driving GSH synthesis. BSO is phosphorylated by ATP on GCLC, and the resulting phosphorylated sulfoximines create strong bonds with the enzyme, ultimately inhibiting GCLC [212]. Preclinical study indicates that BSO’s action inhibits GSH production, leading to increased ROS levels, ultimately triggering apoptotic cell death in neuroblastoma cells [213]. BSO in combination with melphalan was evaluated in Phase I trials to assess the toxic effects, pharmacokinetics, and response rate of patients. For neuroblastoma (NCT00002730 and NCT00005835), the therapeutic outcomes are undisclosed, and for melanoma (NCT00661336), the trial was discontinued for reasons that remain unknown. While BSO exhibits some anti-tumor activity, its clinical investigation is still very limited, necessitating additional research to determine its safety and efficacy in cancer therapy. The repercussions of GSH depletion extend further, as it compromises the functionality of GPX4. GSH depletion sets the stage for ferroptosis induction in hepatocellular carcinoma cells [214]. Collectively, the interplay between GSH, GPX4, and cellular resilience highlights the potential of GSH-targeted strategies to improve the efficacy of standard treatments and possibly alleviate drug resistance in cancer therapy.

#### GPX4 inhibitor

As a central regulator of lipid peroxidation, GPX4 plays a pivotal role in preventing lipid peroxidation-driven cell death by converting lipid ROS into their corresponding lipid alcohols [215]. The pronounced dependence of persistent, drug-resistant malignancies on GPX4 underscores its importance, and its inactivation has the potential to eliminate these cancer cells in vitro and avert tumor recurrence in vivo [216].

The therapeutic landscape has witnessed the emergence of various pharmacological strategies tailored to orchestrating cell death, with a particular focus on triggering ferroptosis by directing their efforts toward depleting GPX4. Among these strategies, small-molecular compounds (e.g., RSL3 and ML162) directly engage GPX4 by covalently binding to selenocysteine, culminating in ferroptosis induction. While these compounds principally target GPX4, they might also exert effects on TXNRD1 in A549 and H1975 cancer cells [217]. The chloroacetamide moiety embedded in the chemical structure of RSL3 and ML162 contributes to their GPX4 inhibitory activity [217]. For example, a co-crystallization strategy reveals that ML162 effectively targets all catalytic tetrad residues in GPX4 by interacting with Sec46, Gln81, Trp136, and Asn137, thereby completely obstructing the active site [218]. Furthermore, ML210 takes the form of diacylfuroxans, which transform into its corresponding α-nitroketoxime, JKE-1674, within cells. Structure–activity relationship studies indicate that potential nitrile oxide species derived from diacylfuroxan bind specifically to the catalytic (seleno)cysteine residue 46 of GPX4 [219]. This derivative aptly interacts with the active site selenocysteine of GPX4, forming the basis for its action [220].

Excitingly, genetic observations suggest that GPX4 is not limited to preventing ferroptosis; rather, it also extends its influence to mitigating other cell death pathways, such as apoptosis, necroptosis, pyroptosis, and parthanatos [48]. Remarkably, increasing evidence also points toward GPX4 protein degradation as a means to incite cancer cell ferroptosis and apoptosis in hepatocellular carcinoma and breast cancer cells [221–223]. These findings align with the idea that GPX4 is involved in sustaining the mitochondrial membrane potential under conditions of oxidative stress [224].

While targeting GPX4 holds promise in cancer treatment, a challenge arises from its widespread expression in both cancer and immune cells, which can potentially cause side effects when using traditional GPX4 inhibitors. A recent study introduced N6F11 as a novel ferroptosis activator that specifically induces TRIM25-dependent GPX4 degradation in pancreatic cancer cells rather than immune cells [223]. However, there are currently no specific GPX4 inhibitors that have entered clinical trials. Overall, the multifaceted role of GPX4 makes it an intriguing focal point for therapeutic interventions and demonstrates its potential to shape the future of cancer therapy.

#### TXN inhibitor

TXN experiences reversible NADPH-dependent reduction facilitated by TXNRD. The concept of TXN inhibitors has emerged as an innovative domain within the realm of anti-cancer agents, imparting the capability to stimulate the generation of ROS. In this context, PX-12 (1-methylpropyl-2-imidazole disulfide) takes center stage as an irreversible inhibitor of TXN1, showcasing remarkable anti-tumor potential. PX-12 achieves a reduction in TXN1 activity either by covalently binding to the key cysteine residue Cys73 in TXN1 or by enhancing the dimerization of its oxidized form [225]. Preclinical studies show that PX-12 impedes hepatocellular carcinoma cell proliferation in vitro and curtails tumor dimensions in mouse models by instigating ROS-dependent apoptosis [226]. Furthermore, PX-12 regulates metastasis of colon cancer cells by diminishing the expression of vascular endothelial growth factor and attenuating hypoxia-inducible factor 1 subunit alpha (HIF1A) levels [227].

A Phase I trial demonstrated that PX-12 administration is safe and well-tolerated in patients with advanced refractory cancers [228]. However, a Phase I clinical involving monotherapy with 24-h intravenous PX-12 for treating advanced gastrointestinal cancer, while completed, showed no clinical activity but exhibited an atypical toxicity profile [229]. Similarly, a Phase II clinical trial assessing PX-12 in patients with previously treated advanced pancreatic cancer was terminated due to a lack of notable clinical efficacy [230]. Therefore, there is an urgent need to develop effective TXN inhibitors for clinical applications.

Intriguingly, a novel compound known as ferroptocide has emerged, functioning as a TXN inhibitor and thereby eliciting ferroptotic cell death in cancer cells [231]. This discovery alludes to the potential anti-ferroptotic role of TXN.

Consequently, TXN inhibitors remain both promising and complex as potential candidates in the fight against cancer.

#### TXNRD inhibitor

TXNRD, a selenium-containing protein, assumes a pivotal role in modulating the delicate balance of thiol redox between the formation and elimination of ROS. In recent years, TXNRD has gained increasing prominence as a pivotal regulator in tumor development, thereby elevating its status as a promising target for innovative cancer treatment strategies [232].

Within the spectrum of utilized inhibitors, auranofin, a gold(I)-containing antirheumatic arthritis drug in clinical use, has prominently emerged. Auranofin has demonstrated the capability to induce oxidative stress and apoptosis by hampering TXNRD activity in lung and breast cancer cells [233, 234]. In preclinical studies of cancer cell lines and tumor xenografts, auranofin exhibits remarkable sensitivity across various forms of drug-resistant cancer cells, including ovarian cancer with a cisplatin-resistant phenotype [235]. Auranofin has entered Phase I/II clinical trials for patients with chronic lymphocytic leukemia (NCT01419691), ovarian cancer (NCT01747798), and lung cancer (NCT01737502), which are still ongoing. Hence, these efforts underscore the importance of TXNRD as a potential therapeutic target for cancer. The mode of action of auranofin hinges on its interaction with the redox-active center of TXNRD, specifically its selenocysteine-containing site, consequently impeding TXNRD function [236]. The X-ray spectroscopy analysis demonstrates the direct and complete binding of the Au atom of auranofin ligand to the Se atom in TXNRD [237]. Furthermore, auranofin can potentially induce paraptosis, an alternative mechanism through which its anti-tumor effects manifest [234]. However, auranofin has several off-target effects beyond its primary target TXNRD. For instance, auranofin also targets or inhibits other proteins, including proteasome and proteasomal deubiquitinases in breast cancer cells [234, 238]. Moreover, resistance to auranofin may occur due to the aberrant expression of multiple drug transporters (e.g., SLC22A1, SLC47A1, SLCO1B1, and ABCBs) in cancer cells [239]. Thus, it is crucial to explore structural modifications of auranofin to reduce drug resistance.

In addition, a slew of emerging classes of TXNRD inhibitors have surfaced, encompassing diverse agents such as natural products, metal complexes, and nitro (hetero) aromatic compounds [240]. In summary, delving further into the potential merits of specific TXNRD inhibitors for cancer treatment holds promise and invites subsequent exploratory endeavors. The multifaceted interactions and impact of these inhibitors within the broader cellular context facilitate the discovery of innovative strategies for combatting cancer.

The increased reliance of tumors on antioxidant systems bestows a potential advantage on oxidative cell death induced by antioxidant enzyme inhibitors compared to conventional treatment modalities. Recently, ferroptosis, a prominent example of oxidative cell death, has garnered significant attention within the scientific community. However, many of these inhibitors, such as RSL3 and ML162, have exhibited suboptimal pharmacological properties in preclinical animal models, while others like BSO and auranofin have not yielded the desired anti-cancer effects in clinical trials, limiting their potential for clinical translation or application. In addition, FSP1 and ACSL4 are critical regulators of ferroptosis, with significant implications for cancer therapy. Inhibitors of FSP1 and inhibition of ACSL4 (e.g., through a high-fat diet) can increase and decrease the sensitivity of cancer cells to ferroptosis, respectively [241, 242]. It is crucial to note that ROS generated by conventional treatment methods may arise from their off-target effects, which adds complexity to the exploration of how oxidative cell death can be optimized for more effective anti-cancer therapies. Accumulating evidence suggests that nanoparticles have emerged as a potent and versatile tool for inducing ROS to achieve therapeutic effects in cancer treatment [243].

## Conclusions and perspectives

The study of oxidative cell death mechanisms in cancer therapy has revealed promising possibilities for innovative treatment strategies. Modifying the delicate balance of ROS in cancer cells is an attractive method for triggering selective cell death through various pathways, such as ferroptosis, apoptosis, necroptosis, pyroptosis, parthanatos, oxeiptosis, and paraptosis. There are several opportunities and challenges associated with targeting oxidative cell death.

Opportunities: (1) selective cancer cell killing: compared to normal cells, tumor cells tend to have higher levels of ROS, largely due to their heightened metabolic activity. This phenomenon is often exploited in cancer treatments, where therapies aim to increase ROS levels beyond a threshold that cancer cells can tolerate, effectively inducing their death while sparing normal cells [9]. To counteract these elevated ROS levels, cancer cells often upregulate ROS-scavenging genes, making them attractive targets for anti-cancer therapies. Key players in this context, including GPX4, SLC7A11, TXN, and TXNRD, have emerged as critical targets. Moreover, the significant implications of NRF2 hyperactivation, KEAP1 mutations, and BACH1 stabilization in cancer initiation, progression, and therapy resistance highlight the promising prospects of targeting the NRF2–BACH1 signaling axis for therapeutic interventions in cancer management. This approach has significant potential, given the unique vulnerability of cancer cells to oxidative stress—a characteristic that can be exploited for therapeutic benefit. The corresponding therapeutic interventions can further increase ROS levels, potentially exceeding the threshold for cell death in tumor cells. Therefore, oxidative cell death mechanisms offer the potential to selectively target and eliminate cancer cells while sparing healthy tissues, thereby minimizing the adverse effects commonly associated with traditional therapies. (2) Persister cancer cells: intratumoral heterogeneity has profound implications for cancer therapy, as different clonal populations within a tumor may respond differently to treatment. With conventional therapeutic agents, certain cancer cells can evade cell death, leading to the persistence of a tumor mass containing tolerant or persister cells. Notably, the use of ROS inducers or GPX4 inhibition can effectively eliminate these persister cancer cells, thereby preventing the development of acquired drug resistance and tumor recurrence in vivo [216, 244]. However, these approaches may increase ROS levels, leading to the accumulation of senescent cells [245], which can be detrimental to the patient. (3) Combination therapies: combining oxidative cell death inducers with conventional treatments, such as chemotherapy and radiotherapy, can synergistically enhance treatment effectiveness, potentially overcoming drug resistance and improving patient outcomes. The combination of ferroptosis inducers with conventional therapy has shown favorable tolerability and minimal toxicity in preclinical models [188]. Additionally, the identification of small molecules, natural products, and novel compounds that target crucial regulators of oxidative stress pathways has expanded the range of emerging therapeutic options. (4) Emerging research areas: cuproptosis, a form of mitochondrial cell death induced by copper overload, is gaining increasing attention as a novel therapeutic target for oxidative cell death in cancer [246]. This emerging focus holds significant potential to revolutionize cancer treatment strategies [247].

Challenges: (1) complexity of ROS regulation: ROS is essential for normal cellular functions. ROS serves as pivotal signaling molecules in cellular physiology, with their levels tightly regulated to maintain cellular homeostasis. Low levels of ROS are crucial for normal cellular functions, including redox signaling, cell proliferation, differentiation, and immune response modulation [11]. For instance, ROS-mediated activation of transcription factors such as NF-κB orchestrates immune responses and inflammatory processes essential for host defense mechanisms [248]. Additionally, ROS acts as secondary messengers in intracellular signaling cascades, modulating cellular processes such as antioxidant gene regulation, cell proliferation and survival, and autophagy [249–251]. However, low to moderate levels of ROS can promote cancer cell survival and metastasis pathways through DNA damage-induced genomic instability, epigenetic regulation, metabolic reprogramming, and generation of pro-inflammatory and pro-tumorigenic microenvironment in a context-dependent manner [8, 12, 13, 26]. In contrast, excessive ROS can eliminate tumors. Therefore, maintaining the right drug dosage to achieve adequate ROS levels may be essential for anti-tumor efficacy. However, the challenge lies in preventing ROS-induced promotion of tumor cell proliferation in specific contexts. (2) Resistance mechanisms: cancer cells can develop alternative mechanisms to counteract oxidative stress, including bolstered antioxidant defenses and modified ROS-scavenging pathways. For instance, tumor cells that overexpress FSP1, a GPX4-independent ferroptosis suppressor, exhibit relative resistance to GPX4 inhibitors. An encouraging strategy involves the development of small molecule inhibitors or alternative therapeutic agents that directly target FSP1, consequently augmenting the susceptibility of tumor cells to GPX4 inhibitors [252]. Targeting autophagy may enhance the efficacy of current cancer therapies by promoting ROS-induced apoptosis [253]. However, blocking autophagy impedes autophagy-dependent ferroptosis in tumor cells, necessitating a more detailed investigation into the types of ROS-induced cell death [254]. Additionally, the consumption of various food-derived antioxidants, such as vitamin C, might interfere with ROS-based anti-cancer strategies. Addressing the challenge of overcoming or preventing inherent or acquired resistance to ROS-based anti-cancer approaches could be a significant concern in the future. (3) Clinical translation: while certain clinical drugs can induce cell death through ROS, the exact extent of the anti-cancer effects of these drugs has not been determined. In contrast, specific oxidative cell death can be triggered by certain preclinical drugs that target antioxidant systems, representing a potential direction for future drug development. Although several preclinical anti-cancer outcomes have been achieved, such as with ferroptosis inducers, translating oxidative cell death mechanisms into effective clinical therapies requires rigorous testing, including assessments of bioavailability, toxicity, and off-target effects. Therefore, the clinical translation of ROS-targeted agents is still in its early stages and necessitates significant progress. To address the challenges mentioned and promote the clinical translation of ROS-targeted agents, we could improve drug absorption and distribution by optimizing the chemical structure of the drug or using delivery systems like nanoparticles. High-throughput screening techniques may help to identify potential ROS-targeted drugs. In addition, the development of personalized treatment strategies based on individual patient profiles could amplify the efficacy of cancer therapeutics.

In conclusion, advancements in understanding oxidative cell death mechanisms could reshape the field of cancer therapy. The interplay among ROS, antioxidant networks, and cell death pathways reveals a complex yet promising landscape. As scientific research has delved deeper into this landscape, precision-oriented strategies for targeted cancer treatments come into view. However, the path to clinical application requires well-organized exploration through multidimensional research and multidisciplinary approaches.
